# RSSI-Based Smooth Localization for Indoor Environment

**DOI:** 10.1155/2014/639142

**Published:** 2014-06-29

**Authors:** Yujian Wang, Bin Zhao, Zhaohui Jiang

**Affiliations:** IPPFRD, Alcatel-Lucent Shanghai Bell Co. Ltd., Shanghai 201206, China

## Abstract

Radio frequency (RF) technique, for its better penetrability over traditional techniques such as infrared or ultrasound, is widely used for indoor localization and tracking. In this paper, three novel measurements, point decision accuracy, path matching error and wrong jumping ratio, are firstly defined to express the localization efficiency. Then, a novel RSSI-based smooth localization (RSL) algorithm is designed, implemented, and evaluated on the WiFi networks. The tree-based mechanism determines the current position and track of the entity by assigning the weights and accumulative weights for all collected RSSI information of reference points so as to make the localization smooth. The evaluation results indicate that the proposed algorithm brings better localization smoothness of reducing 10% path matching error and 30% wrong jumping ratio over the RADAR system.

## 1. Introduction

Nowadays, location is the important information for many innovative applications [1]. For example, smart asset management (SAM) system requires the position of each asset. As the asset is moved from one place to another, the manager should master the physical movement in a real-time manner. With location information, the system helps to reduce the searching time of each asset and improve its usage efficiency for the enterprise. Obviously, it is of significant importance to determine the real-time position of each mobile entity (e.g., an asset), which is called* indoor localization or tracking*.

The techniques for indoor tracking are classified into two broad categories: non-RF and RF techniques. In the former category, non-RF methods, such as infrared or ultrasound, may be used alone or together with RF for indoor tracking [2, 3]. From the view of marketing, the additional equipments, such as accelerometer, compass, and gyroscope, increase the cost of the large-scale deployment. Besides, these techniques may not work well in the complicated indoor environment for its worse penetration. For instance, the signals will be blocked when the devices are buried in the users' wallets or bags [1].

As RF measurement (e.g., RSSI) will be easily obtained during the wireless communication, there are many RSSI-based methods for indoor localization. Previous work mostly cares about the position error, which denotes the average distance between the real position and located position. However, this measurement cannot fully reflect the performance for continuous localization in practice. For example, the entity is moving from point *A* to point *B*. Whereas, as the instability and unreliability of RSSI, the obtained tracking path is *A*-*C*-*B*, though point *C* does not lie between point *A* and point *B*. In this case, though the one-shot wrong jumping may not greatly increase the average position error, this will result in wrong path determination. So that the users may regard the localization result as unreasonable for the wrong tracking path. To express the tracking efficiency, we call it smooth localization if the computed path by the algorithm is exactly the same as the real path for a mobile entity. However the multipath reflection and wireless interference bring some challenges to implement the smooth localization. (1) RSSI is not stable. The value of RSSI is determined by the transmission power, distance, transmission path, and so forth. Even between two fixed nodes, the RSSI value varies as time goes by. (2) RSSI is not always reliable. Transmission reliability will change under different scenarios. When the number of mobile entities is increasing, the communication interference increases, which reduces the transmission reliability. (3) There is no causal relationship between RSSI and Euclidean distance, so that large distance estimation is brought about by judging from RSSI information only.

The researchers have designed many algorithms to conquer one or some of the above challenges for indoor localization. For example, as there is no apparent function between RSSI and Euclidean distance, several works [4] compute the nodes' localizations based on the connectivity information. Then, RSSI is used to enhance the location precision [5, 6]. However, there is mere work dealing with the unreliability and instability of RSSI, which make many algorithms unfit for the practical applications. The existing RF-based tracking systems mostly focus on the precise position or average position error. However, no apparent performance measurement has been studied for smooth localization. For example, the MERIT system [7] defines a new measurement for indoor localization, called area decision accuracy, to determine where the entity locates by comparing the average RSSIs of different areas. As RSSI is not always stable or reliable, it does not work well under the indoor environment. MERIT [7] requires the denser AP deployment and does not fit for the areas with different area sizes. As in the SAM application, wrong area decision will result in wrong asset searching and asset statistics. With the negative features of RSSI, smooth localization becomes an extremely difficult challenge for researchers. As we know, there is no special work aiming to smooth localization. The main contribution of this paper is as follows.

- Three novel measurements, point decision accuracy, path matching error, and wrong jumping ratio, are defined to express the localization smoothness for indoor environment.
- A RSSI-based smooth localization (RSL) algorithm is proposed for the indoor localization. The algorithm first selects some reference points and constructs a connected graph for these points based on the architectural features. Then, reference points will be assigned weights and accumulative weights by the collected RSSI information. Finally, a novel tree-based mechanism is designed to estimate the moving track with high probability for smooth localization.
- The proposed algorithm is validated on the WiFi test bed. The evaluation results indicate that the proposed algorithm can get better localization smoothness compared with the previous works. For example, the RSL algorithm can reduce the path matching error and wrong jumping ratio by about 10% and 30% over the RADAR system.

In Section 2, the localization framework is introduced, and three novel performance measurements for smooth localization are defined. The RSSI-based smooth localization algorithm is discussed in detail in Section 3. The validation is described in Section 4 according to the above measurements. Finally, the conclusion is drawn in Section 5.

## 2. Localization Performance Measurement

### 2.1. Localization Framework

Many algorithms of previous work may locate the mobile entity to arbitrary positions in the field. However, as RSSI information is unstable and wireless transmission apt to be unreliable, the traditional RSSI-based methods might result in unstable localization. That is, the localization result will jump from one point to another, while the entity is stationary to a fixed spot. To provide smooth localization, this procedure mainly consists of two steps: (1) RSSI sampling and (2) position determination. Like the previous method [8], the localization framework first selects many reference points (also called representational points) in the field. For example, the central point of a room will be selected to represent this room. The system samples the RSSI information on these discrete points for a durative period. In the runtime, the mobile entity will be located on some presampled positions by the collected RSSI information. In the second step, different mechanisms are designed to improve the localization performance. The main advantage of this framework is to increase the localization efficiency and stability compared with the previous mechanisms [7]. The task of this paper is to design efficient methods to improve the localization smoothness under this practical framework.

### 2.2. Localization Measurement Definition

**Figure 1:**
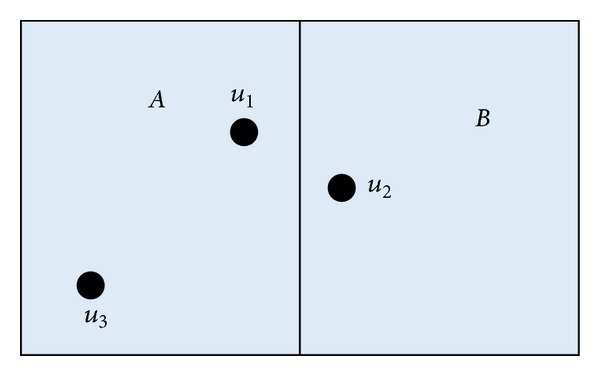
Illustration of localization measurement.

The previous algorithms [5, 7–9] mostly take the average position error, which denotes the distance between the real position and located position, as the main performance measurement. However, this measurement cannot often reflect the real effect for indoor localization. For example, there are two rooms in a field, shown in Figure 1. Assume that the current position of a mobile entity is *u*_1_. Two localization algorithms obtain the different position results, denoted by and through RSSI information. From the view of average position error, position *u*_2_ is closer to *u*_1_ than to *u*_3_, so that theoretically the average position error between *u*_1_ and *u*_2_ is smaller than that of *u*_1_ and *u*_3_. However, *u*_1_ is located in the same room as *u*_3_. Thus, it is more reasonable to locate the mobile entity on position *u*_3_ compared to position *u*_2_.

In the following, we will define some practical performance measurements for indoor localization. There are two different scenes for indoor localization. One is static, the other is mobile.

For static scenes, the entity mostly stays at one position or around for a long time. Under the above localization framework, each position in the field will be logically appointed to a reference point. For example, two points are in the same area or close to each other. We adopt the* point decision accuracy *(PDA) to measure the localization efficiency under the static scenes. In this case, point decision accuracy is defined as the correct probability that the mobile entity is located at the appointed reference point by the localization algorithm. For example, as shown in Figure 1, we regard that each point in the area *A* is appointed to position *u*_3_, for they lie in the same room. The system collects the RSSI information for 100 time slots as the mobile entity is on point *u*_1_. If there are 90 time slots in which the localization result is *u*_3_, we regard that the point decision accuracy of this algorithm is about 0.9. If each area only contains one reference point, point decision is the same as area decision [7]. Thus, PDA is more general than area decision accuracy.

For mobile scenes, the entity continuously moves from one position to another. However, one may stay on a certain point for a short time compared with a long period. To reflect the effect of the practical movement, we will define two measurements to express the localization smoothness. One is called as* path matching error* (PME). We consider the discrete-time division. Assume that entity *u* locates on a point *p*(*t*) in time slot *t*. Note that, the mobile entity may not locate on a reference point. In comparison, as each position will be logically appointed to a reference point in the field, we use the appointed reference point to denote the real position. That is, *p*(*t*) is a reference point, to which the real position of entity *u* may be appointed. Then, a real path can be described as a position string RP = *p*(1),…, *p*(*n*), where *n* is the total number of time slots. After information processing, the server may obtain the localization result *r*(*t*) in time slot *t*. Note that, *r*(*t*) is a reference point under the above localization framework. Thus a localization result path denoted by LP = *r*(1),…, *r*(*n*) is obtained. To express the similarity between two paths, we compute their maximum common substring of two paths RP and LP, denoted by MCS(RP, LP). Moreover, its length is denoted by |MCS(RP, LP)|. Then, PME is calculated as

(1) $$\begin{array}{c} \text{PME}\left(\text{RP},\text{LP}\right)=1-\frac{\left|\text{MCS}\left(\text{RP},\text{LP}\right)\right|}{n}. \end{array}$$

The other measurement is called* wrong jumping ratio* (WJR). The unstable and unreliable RSSI information will result in wrong jumping, which greatly debases the localization. Assume that the real track of a mobile entity is “1133.” With RSSI information, the localization result is “1123.” So, there is one wrong jumping from point 1 to point 2. Note that, this jumping will result in wrong path computation. WJR is defined as the average number of wrong localization jumping during 100 time slots. For example, the system collects the RSSI information and computes the localization for *n* time slots. The number of wrong localization jumping is *m*. Then, WJR is defined as

(2) $$\begin{array}{c} \text{WJR}=\frac{m}{n}\times 100. \end{array}$$

The smooth localization aims to reduce the path matching error and to avoid the wrong localization jumping. Now, we give examples to illustrate the definitions of PME and WJR. For example, the real path consists of some discrete positions and is expressed by RP = “1112233345.” Assume that the first algorithm derives the path as LP_1_ = “1112223345.” By the definition, the maximum common substring of RP and LP_1_ is “111223345,” and its length is 9. So, its path matching error is 0.1. Though LP_1_ cannot fully be matched to RP, there is no wrong jumping between two paths. Thus, the WJR of LP_1_ is 0. This algorithm just locates the entity to point 3 with a delay. Assume the second algorithm obtains a path LP_2_ = “1142223345.” Similarly, the maximum common substring of RP and LP_2_ is “11223345,” and its length is 8. As a result, its path matching error is 0.2. Observing the localization result LP_2_, there is one wrong localization jumping from point 1 to point 4, and its WJR is (1/10) × 100 = 10. The third algorithm obtains a path LP_3_ = “1142233245.” The maximum common substring of RP and LP_3_ is “11223345,” and its length is 8. Then, its path matching error is 0.2. However, there are two wrong jumping, from point 1 to point 4 and from point 3 to point 2, respectively. Its WJR is (2/10) × 100 = 20. According to the above description, we regard that LP_1_ is much smoother than paths LP_2_ and LP_3_. Though LP_2_ and LP_3_ reach the same PME, LP_2_ will work better than LP_3_ for WJR.

## 3. Smooth Localization Algorithm Description

In this section, we will design a RSSI-based smooth localization (RSL) algorithm, which aims to obtain higher point decision accuracy, lower path matching error, and lower wrong jumping ratio. The RSL algorithm consists of three steps, reference point selection, graph construction, and localization determination, respectively.

### 3.1. System Equipments

Before algorithm description, we first introduce the main equipment in the system.

**Figure 2:**
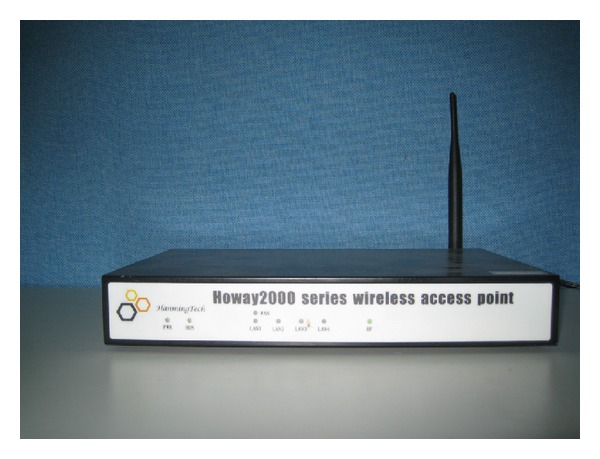
Access point.

**Figure 3:**
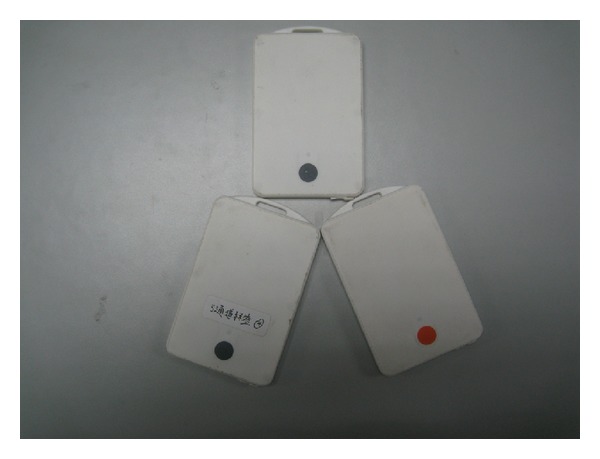
WiFi-compatible card.

There are two main categories of equipments in the system. One is access point (AP), shown in Figure 2. Each AP can support the standard 802.11 b/g/n and is used to collect the detection packets from all the mobile entities in each time slot. Moreover, all access points can form a wireless backbone network and transmit the obtained RSSI information to the localization server. As WiFi networks are widely deployed, the localization system can be built on these wireless networks, which helps to save deployment costs. The other is WiFi-compatible card, shown in Figure 3. Each mobile entity will take a card, which locally broadcasts the detection packets to the neighboring access points. In the system, each card is only capable of wireless transmission, which is energy efficient, cheap, and fit for large-scale applications. After RSSI collection, the localization server will determine the real-time position for each entity.

### 3.2. Reference Point Selection and RSSI Sampling

**Figure 4:**
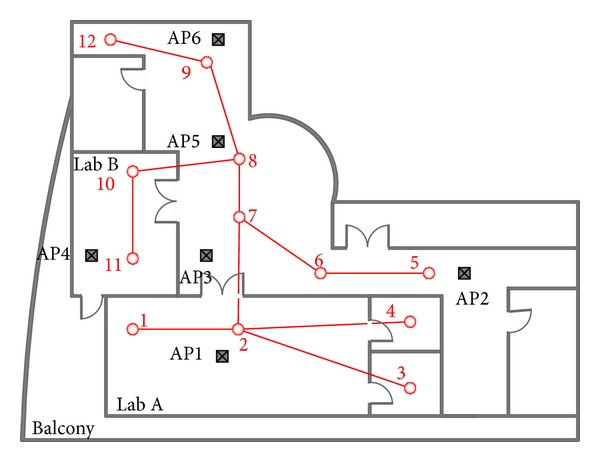
Reference point selection and graph construction.

In this subsection, we first describe the rule for reference point selection in the target field. Inside the building, the fixed structures have formed the natural partitions, such as offices, meeting rooms, and aisles. Generally, a representational position will be selected in each area, such as points 3 and 4 in Figure 4. However, there are some rooms with a larger size. We will choose multiple reference points which are uniformly distributed in one room, such as points 10 and 11 in Figure 4. As the RSSI information varies much more in a large room, the selection of multiple reference points may help to improve the localization accuracy and efficiency. After selection of reference points, we sample the RSSI information on each reference point for a durative period, such as 30 minutes, and keep the sampling results into the localization database on the server. To save the time, multiple cards are used to sample the RSSI information on several reference points simultaneously. For a certain access point AP_*i*_, the average RSSI value from the reference point *j* is denoted by *μ*_*ij*_, and its variance is *σ*_*ij*_.

Note that, in the underground mine environment, the working field is mostly a linear area. If a long corridor is regarded as one area, the relative distance from the entity to AP might be large. To solve this, we divide the linear laneway into some “virtual areas,” in which the center position will be chosen as the reference point of this virtual area. The sparse division will decrease the localization accuracy, and the dense division may result in serious localization jumping, thus decreasing the localization smoothness. Given a long laneway, we deploy two access points with a distance of 100 m. Our testing shows that it is an efficient way to divide this laneway with 100 m into 5 virtual areas, where the length of each area is 20 m. In this way, the localization algorithm will satisfy both accuracy and smoothness.

### 3.3. Graph Construction for Reference Points

In this subsection, a connected graph *G* will be constructed for the reference points *V*, which is used to determine the track of each mobile entity and to improve the localization smoothness. According to the description, each reference point *u* belongs to a certain partition or area, denoted by *A*(*u*). In the following, *u* and *v* are two reference points. We describe the rules for graph construction.

1. *A*(*u*) and *A*(*v*) are two areas; that is, *A*(*u*) ≠ *A*(*v*). There are three cases to be discussed.
  - *A*(*u*) and *A*(*v*) are not connected or shared with a door. Two points are not connected either.
  - *A*(*u*) and *A*(*v*) are connected or shared with a door. Moreover, points *u* and *v* are the shortest point pair between two connected areas. Two points are connected.
  - *A*(*u*) and *A*(*v*) are connected or shared with a door. However, points *u* and *v* are not the shortest point pair between two connected areas. Two points cannot be connected.
2. *A*(*u*) and *A*(*v*) are the same area; that is, *A*(*u*) = *A*(*v*). Two points are also connected in the graph *G*. Generally, if there are more than two reference points in one area, they are all connected with others.

In this way, we have constructed an undirected graph *G*, illustrated in Figure 4. By rule 1-a, point 3 cannot be connected with point 7, for two areas are not connected. By rule 1-b, point 3 is connected with point 2. However, point 3 cannot be connected with point 1 according to rule 1-c. Followed by rule 2, point 10 and point 11 are connected, for they belong to the same area.

### 3.4. Tree-Based Smooth Localization

As described above, the time is divided into many discrete slots. For example, the length of each time slot is 1 second. During each time slot, the mobile entity will locally broadcast the detection message. The neighboring access points capture the detection message, compute the RSSI values, and transmit to the server. As a result, the server can obtain the RSSI information (or RSSI vector) about each mobile entity in this time slot. This vector can be expressed as {(AP_*i*_1__, RSSI_*i*_1__),…, (AP_*i*_*s*__, RSSI_*i*_*s*__)}, where each item (AP_*i*_*j*__, RSSI_*i*_*j*__) denotes that access point AP_*i*_*j*__ has detected the RSSI value of RSSI_*i*_*j*__ from the mobile entity, and *s* is the number of RSSI values in the vector.

The previous works [8, 10] have proposed a RSSI-distance method to measure the matching similarity between the current RSSI vector and each sampled RSSI vector in the localization database. However, RSSI information between two fixed nodes is not stable as time passed and wireless transmission is apt to unreliable, which may lead to the incorrect localization result, worse path matching error, and wrong jumping ratio. Next, we will design a tree-based smooth localization (TSL) mechanism for practical indoor tracking. This subalgorithm contains three steps, weight assignment, accumulative weight computation, and localization determination.

In the first step, each reference point will be assigned a weight by RSSI information. For the mobile entity *u*, assume that the RSSI information detected by AP_*i*_ in current time slot is denoted by *x*_*i*_. It is also assumed that the RSSI information obeys the Poisson distribution [11]. Thus, the probability that this entity locates on the position *j* from the view of AP_*i*_ is

(3) $$\begin{array}{c} P_{ij}=\frac{1}{\sigma_{ij}\sqrt{2\pi }}e^{-{\left(x_{i}-\mu_{ij}\right)}^{2}/2\sigma_{ij}^{2}}, \end{array}$$

where the constants *μ*_*ij*_ and *σ*_*ij*_ are introduced in the above subsection. The AP set that receives the RSSI information from mobile entity *u* in the time slot *t* + 1 is denoted by {AP_*i*_1__, AP_*i*_2__,…, AP_*i*_*s*__}, where *i*_1_, *i*_2_,…, *i*_*s*_ ∈ [1, *m*], and *m* is the number of access points in the system. The combination probability that the entity locates on the reference point *j* is denoted by *P*_*j*_ as

(4) $$\begin{array}{c} P_{j}=\overset{s}{\underset{k=1}{\prod }}P_{i_{k}j}. \end{array}$$

With the gathered RSSI information during the time slot *t* + 1, we assign a weight *w*(*j*) for each reference point *j*, which denotes the possibility that the mobile entity *u* may locate on this point with the collected RSSI information. The weight of each area is computed as

(5) $$\begin{array}{c} w\left(j\right)=\frac{P_{j}}{\sum_{k=1}^{n}P_{k}}, \end{array}$$

where *n* is the number of reference points in the system. By this way, each reference point has been assigned a weight. Note that, the weight assignment method is not unique. We also can use another method for weight assignment. Though the weight is not strictly accurate, this is useful to predict the moving track.

The second step of TSL will compute the accumulative weight for each reference point on the constructed tree. For simplicity, *L*(*t*) denotes the localization result of time slot *t* for the mobile entity *u*. To determine the localization in time slot *t* + 1, the algorithm constructs a width-first searching (WFS) tree *T* rooted at the point *L*(*t*) for graph *G*. Each point *j* knows its children set, denoted by chl(*j*). We compute the accumulative weight for point *j*, denoted by Aw(*j*), as follows. For each leaf point in tree *T*, the accumulative weight is its weight. For each intermediate point, the accumulative weight is the sum of accumulative weights of all the children nodes plus its weight. That is,

(6) $$\begin{array}{c} \text{Aw}\left(j\right)=\{\begin{array}{cc} w\left(j\right), & j\;\text{is}\;\text{a}\;\text{leaf}\\ w\left(j\right)+\underset{k\in \text{chl}(j)}{\sum }\text{Aw}\left(k\right), & \text{else}. \end{array} \end{array}$$

Finally, the TSL algorithm will search for a reference point whose accumulative weight is more than a threshold *w*_0_ and furthest from the root, denoted by *j*_0_. Note that, the distance of two nodes is defined as the hop number between two nodes in the tree *T*. As a result, the mobile entity will locate on the point *j*_0_ in time slot *t* + 1. That is, *L*(*t* + 1) = *j*_0_. This indicates that the mobile entity has moved to the reference point *j*_0_ in time slot *t* + 1 with very high probability. If there is no point whose accumulative weight is more than *w*_0_, we regard that the tracked entity is static during time slot *t* + 1. That is, *L*(*t* + 1) = *L*(*t*). Though the entity may move in this time slot, the system cannot correctly judge the moving track as the RSSI information is unstable. The system will take a localization decision with a delay. As a result, the wrong localization jumping among several reference points will be avoided as possible. The TSL subalgorithm is formally described in Algorithm 1.

### 3.5. Illustration of the RSL Algorithm

**Figure 5:**
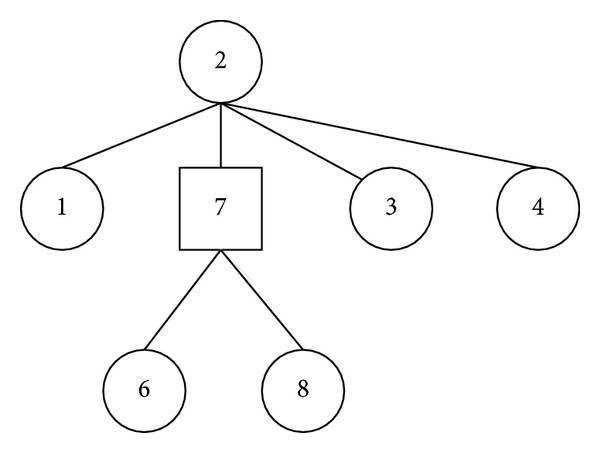
Illustration of localization smooth.

We illustrate the RSL algorithm by an example. The reference points are chosen and drawn in Figure 4. The parameter *w*_0_ is 0.50. For time slot *t*, assume that *L*(*t*) = 2. To compute the localization of time slot *t* + 1, the algorithm first constructs a WFS tree rooted at point 2, shown in Figure 5. Assume that the mobile entity is currently located around the reference point 8. After RSSI collection, we will compute the weight and accumulative weight of each point. By Table 1, the accumulative weights of point 6 and point 8 are 0.20 and 0.25, respectively. The accumulative weight of point 7 is the sum of weights of points 6, 7, and 8. So, Aw(7) = 0.20 + 0.10 + 0.25 = 0.55. According to the RSSI information, the algorithm may not distinguish the real-time position, either point 6 or point 8, for the mobile entity. However, the system can regard that the mobile entity has moved to point 7 with very high probability. We think it is reasonable, for point 7 to lie on the moving path from point 2 to the current position (i.e., point 8).

#### 3.5.1. Algorithm Discussion

When locating the mobile entity in each time slot, the algorithm computes the weights and accumulative weights for all the reference points. As the numbers of reference points and mobile entities are both large, the requirement of computational capacity is very high for the practical localization system. To improve the computation efficiency, we propose a local searching mechanism for the RSL algorithm. Given a maximum velocity of the mobile entity, each entity will not move a long distance in a short time (such as 1 s, 2 s, etc.). For time slot *t* + 1, the RSL algorithm will construct a local tree *T*′ whose maximum depth is not more than *k* rooted at *L*(*t*), where *k* is predefined constant (e.g., 5 or 10) in the system.

## 4. Experimental Results

This section presents the numerical results to demonstrate the efficiency and smoothness of the proposed localization algorithm. Though there are some localization algorithms based on RF techniques, they all require the additional conditions for indoor localization. For example, LANDMARC requires the dense deployment of beacon nodes, and the EZ algorithm [12] will occasionally fix the localization by GPS. Moreover, there is no special work on smooth localization. As a result, we mainly evaluate the performance of the RSL algorithm by comparing with the RADAR system [8] on the WiFi test platform. The RADAR system introduces two different methods of weight assignment, Euclidean distance, and Manhattan distance. So, we will denote RAD + Euc and RAD + Man to express the localization methods with different weight assignments. The experiments mainly observe the performance of three localization measurements, point decision accuracy, path matching error, and wrong jumping ratio, respectively. The definitions for these measurements are described in the above section. The accumulative weight threshold *w*_0_ is set as 0.50. Besides, this proposed algorithm has little effect on the layout of the goods around the mobile entity regarding the spatial uniqueness of RSSI distribution.

### 4.1. Experiment Environment

The experiment is conducted at the Demo Center of Alcatel-Lucent Shanghai Bell Co. Ltd. There are totally 12 reference points and 6 access points in an area of about 400 squares meters. The reference points and AP deployments are also illustrated in Figure 4.

### 4.2. Numerical Results for Localization Algorithms

The first experiment mainly observes the performance of point decision accuracy for different algorithms. In particular, the mobile entity will statically locate in one place for continuous 115 time slots in the evaluations. By the collected RSSI information, we can compute the point decision accuracy for different algorithms.  Table 2 gives the PDA comparison of different algorithms. On the average, the RSL algorithm improves the PDA by about 15% and 8% compared to the RAD + Euc and RAD + Man algorithms. Considering the highlighted worst case, RSL can enhance the worst PDA from 0.54 to 0.69. Thus, the proposed algorithm can get smoother localization compared to the RADAR system for the static case.

The second experiment observes the performance of path matching error and wrong jumping ratio for different algorithms. We select two different paths in the target field. One is 1-2-7-8-9-12, denoted by path A. The other is 5-6-7-8-10-11, denoted by path B. Moreover, the evaluations adopt two different moving patterns through each path. One is moving with the uniform velocity, denoted by pattern a. The other is similar with case a, except that the mobile entity will stay on each reference point for 10 seconds, denoted by pattern b. We give the evaluation results in Table 3 to Table 6, in which the numbers in the brackets denote the lasting time for different paths. For pattern a, the proposed algorithm reduces the path matching error of about 9.2% compared to the RADAR system. Moreover, the RSL algorithm decreases the wrong jumping ratio by at least 30% compared to the RADAR system from Tables 3 and 4. For moving pattern b, the RSL algorithm will reduce the path matching error of about 15.1% compared with the RADAR system. At the same time, the proposed algorithm also decreases the wrong jumping ratio by about 65% compared with the RADAR system from Table 5 and Table 6. That is due to the tree-based mechanism adopted in RSL, which helps to avoid the wrong jumps in localization as much as possible.

Based on the evaluation results, the proposed algorithm can improve three localization measurements compared with the previous algorithms, such as RAD + Euc and RAD + Man. In particular, the RSL algorithm will work excellently for the performance of wrong jumping ratio. As a result, this algorithm improves the localization smoothness compared with the previous algorithms intuitively.

## 5. Conclusion

In this paper, a novel RSL algorithm is designed, implemented, and validated. This algorithm purely depends on RF technique and uses a serial of access points to track the mobile entities in the indoor environment. The evaluations demonstrate that this system is more effective than the previous related works. On our evaluation, the RSL algorithm can reduce the path matching error and wrong jumping ratio by about 10% and 30% compared with the previous systems. As the RSSI information is unstable, RSL may not be fully smooth. In the future, our team will continue to improve the point decision accuracy, path matching error, and wrong jumping ratio. In many applications, delay is another important measurement for localization. In the future, we will study the tradeoff between the smoothness and delay for real-time localization.

## Figures and Tables

**Algorithm 1:**
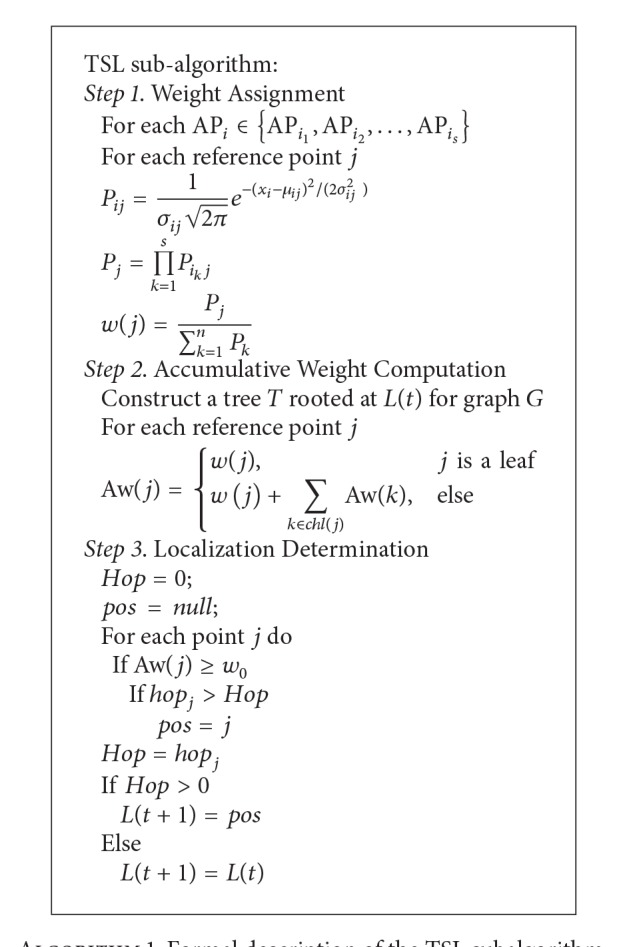
Formal description of the TSL subalgorithm.

**Table 1:** An example of weights and accumulative weights.

Point	1	3	4	6	7	8
*W*	0.10	0.10	0.15	0.20	0.10	0.25
Aw	0.10	0.10	0.15	0.20	0.55	0.25

**Table 2:** PDA comparison for different algorithms.

Number	Real position	RAD + Euc	RAD + Man	RSL
1	1	0.91	0.91	0.91
2	1	0.92	0.92	0.94
3	1	0.68	0.81	0.93
4	7	**0.54**	**0.62**	0.71
5	7	0.77	0.77	0.83
6	9	0.79	0.83	0.87
7	9	0.71	0.70	0.74
8	11	0.64	0.72	0.84
9	11	0.55	0.67	**0.69**

**Table 3:** Performance for path A under pattern a (64 s).

Number	Measurement	RAD + Euc	RAD + Man	RSL
1	PME	0.36	0.36	0.30
2	WJR	20.31	21.88	9.38

**Table 4:** Performance for path B under pattern a (61 s).

Number	Measurement	RAD + Euc	RAD + Man	RSL
1	PME	0.61	0.57	0.57
2	WJR	18.03	16.39	11.48

**Table 5:** Performance for path A under pattern b (109 s).

Number	Measurement	RAD + Euc	RAD + Man	RSL
1	PME	0.43	0.45	0.39
2	WJR	29.36	29.36	10.09

**Table 6:** Performance for path B under pattern b (104 s).

Number	Measurement	RAD + Euc	RAD + Man	RSL
1	PME	0.38	0.42	0.30
2	WJR	25	27.88	8.65
